# Smartphone-Based Gait Assessment Captures Functional Recovery Following Total Knee Arthroplasty

**DOI:** 10.3390/s26020432

**Published:** 2026-01-09

**Authors:** Celeste A. Thai, Jakob R. Marrone, Lauren C. Tran, Britta Berg-Johansen

**Affiliations:** 1 Physical Therapy Department, College of Health Professions, Rosalind Franklin University of Medicine and Science, North Chicago, IL 60044, USA; 2 Biomedical Engineering Department, College of Engineering, California Polytechnic State University, San Luis Obispo, CA 93407, USA; jrmarron@calpoly.edu (J.R.M.); ltran114@calpoly.edu (L.C.T.); bbergjoh@calpoly.edu (B.B.-J.)

**Keywords:** TKA, biomechanics, motion capture, smartphones, gait

## Abstract

Novel smartphone-based methods offer an accessible and promising alternative to traditional tools for performing clinical gait assessments in total knee arthroplasty (TKA) patients. The OneStep app uses the smartphone’s sensors and proprietary machine learning algorithms to measure gait parameters of walking trials, including stride length, step length, step width, gait velocity, cadence, and double stance time. The objectives of this study were to (1) validate the reliability of the OneStep app against a traditional motion capture (MoCap) system and (2) use the OneStep app to measure functional recovery of TKA patients pre- and post-operatively. For Objective 1, walking trials using both OneStep and MoCap were conducted with N = 17 healthy adults (9M/8F, aged 22.29 ± 2.08 years). Results showed that of all gait variables, cadence (*p* < 0.0001) and gait velocity (*p* < 0.0001) exhibited the strongest correlations between methods indicated by their linear regression results, and step width had the weakest correlation between methods (*p* = 0.67). For Objective 2, OneStep gait measurements were collected for N = 11 TKA patients (5M/6F, aged 70.91 ± 6.56 years) at their pre-operative, 2-weeks post-operative, and 6-weeks post-operative appointments. Results showed marked declines in gait properties (decreased stride length, step length, cadence, and gait velocity and increased step width and double stance time) of participants relative to pre-operative values at 2-weeks pre-operative, and an increase/surpassing of pre-operative gait measurements 6-weeks post-operative. The greatest differences were observed in gait velocity between pre-operative and 2-weeks post-operative (*p* = 0.011) and 2-weeks post-operative to 6 weeks post-operative (*p* = 0.005).

## 1. Introduction

Knee osteoarthritis (OA) is a degenerative articular joint disease and the most common type of arthritis diagnosed in the elderly. Roughly 10% of individuals 60 years and older have symptomatic knee OA, and this number can rise to 40% for individuals older than 70 years [1]. Primary knee OA often occurs without any specific apparent event and is typically the result of wear and tear that leads to the progressive loss of articular cartilage. When conservative treatment methods for knee OA are no longer effective, total knee arthroplasty (TKA) is the most common surgical intervention for end-stage knee OA [1,2].

The goals of TKA are to relieve pain, restore the patient to their pre-diseased kinematic alignment, and improve functional status [3]. However, despite proper kinematic alignment and improved self-reported outcomes, patients post-TKA often continue to exhibit gait dysfunction, including reduced cadence, stride length, and gait speed [2].

Gait analysis can be performed to quantitatively and objectively monitor functional recovery of patients following TKA to guide rehabilitation strategies. Motion capture systems are the current gold standard for measuring gait but pose specific limitations. For one, the required equipment for motion capture systems is bulky, expensive, requires significant time investment for calibration and analysis, and is only accessible in motion analysis laboratories, thus limiting the direct translation to clinical practice and real-world monitoring [4,5]. Furthermore, motion capture in a laboratory environment does not simulate natural walking surfaces typically encountered in one’s daily activities [4,6]. On the other hand, inertial measurement unit (IMU)-based motion capture technology is a rapidly growing field that uses sensors to measure motion and orientation data using accelerometers, gyroscopes, and magnetometers. Advantages of IMUs compared to a motion capture system are portability, lower costs of equipment, and suitability for outdoor use.

Smartphones with embedded IMUs are an emerging instrument for gait evaluation, with smartphone-based studies reporting moderate to strong validity and reliability of spatiotemporal gait parameters (e.g., gait velocity, cadence, stride length) for normal walking of healthy adults [4,5,7]. Compared to motion capture systems, smartphones are ubiquitous, cost-effective, portable, possess a user-friendly interface, and do not require specific placements of markers or sensors on the body [5,7,8,9]. The built-in sensors of smartphone IMUs include a 3D accelerometer, 3D gyroscope, and a digital compass [8,10]. Various apps have been built to support gait analysis, which enables patients and clinicians to monitor the functional recovery of post-TKA. One such app, the OneStep app, uses the smartphone’s sensors to create a gait report that includes standard biomechanical measurements of spatial parameters (stride length, step length, step length asymmetry, step width) and temporal parameters (double support, stance, stance asymmetry, double support asymmetry) used in gait assessments. For this study, we analyzed stride length, step length, step width, cadence, gait velocity, and double support time because these gait metrics are often evaluated to assess post-TKA recovery and gait-related declines during aging [2,8,9]. Furthermore, OneStep was chosen for this study based on its promising results for accurately measuring gait metrics in previous studies [4,5].

Gait analysis research using motion capture or smartphone-based assessments have been restricted to limited walking conditions and are also primarily performed on healthy adults. As a result, there are limited gait studies on post-TKA patients; the studies that do focus on the TKA population have relied heavily on patient-reported outcome measures (PROMs) to assess functional recovery. The validity of PROMs results is dubious, as TKA patients have been documented to overestimate their physical activity by as much as 50% [2,9]. For these reasons, smartphone-based gait analysis proves to be a promising rehabilitation technology for a largely unresearched population such as post-TKA patients [4,5,7,11]. Smartphone-based assessments benefit TKA patients by having the ability to objectively monitor their gait recovery pre- and post-TKA during daily activities, share their gait metric data with healthcare providers, and better understand their recovery rates post-TKA.

The purposes of this study were to provide validation of the OneStep app and to determine whether the app is able to detect the expected differences in gait metrics of TKA patients post-operatively. The first hypothesis of this study was that gait analysis using the OneStep smartphone app is valid and reliable compared to a motion capture system (tested in Objective 1). The second hypothesis was that there would be meaningful differences in gait parameters as measured by a smartphone for TKA patients at 2- and 6-weeks post-operative vs. pre-operative (tested in Objective 2).

## 2. Materials and Methods

### 2.1. OneStep Setup

OneStep gathers linear acceleration and angular velocity at 100 Hz to calculate spatiotemporal variables using the embedded sensors of the smartphone and proprietary machine learning gait algorithms [4]. For both the validation and clinic experiments (Section 2.2, Section 2.3 and Section 2.4), the “1-min walking test” with the “phone in the pocket” options were selected in the app. For each walking trial, the smartphone (iPhone 11, Apple Inc., Sunnyvale, CA, USA) was placed vertically in the pocket of the participant’s dominant leg, and the same model was used for all participants of this study. The vertical placement of the smartphone was visually verified prior to data collection. The z-direction of the phone was arbitrary for experiments.

If participants did not have pants with pockets, a magnetic phone holder (Running Buddy, purchased via Amazon) measuring 6.5″L × 1″W × 4″H was used as a simulated pocket. The holder was attached on the waistline of the participant’s clothing as close as possible to where a front pocket would be located.

### 2.2. Validation Experiment Methods

All experiments involving human subjects were approved by the Cal Poly Institutional Review Board and were designed to minimize risk to participants.

To address the first hypothesis, the validation and reliability of OneStep was compared to a motion capture system (MoCap) through validation experiments conducted on 17 healthy adults (9M/8F, aged 22.29 ± 2.08 years, 15 right leg dominant and 2 left leg dominant) sampled from California Polytechnic State University, San Luis Obispo. This range of participants was chosen to reflect similar sample sizes of similar research studies [4,5]. The inclusion criteria were college-aged students aged 18–30 years and English speaking. The exclusion criteria were history of cardiovascular, respiratory, or metabolic disease, history of orthopedic pathology, medical conditions that prohibit physical activity, or pre-existing joint injuries that impact walking ability. The validation phase was conducted in healthy adults to establish the fundamental accuracy and consistency of the OneStep system compared to MoCap before applying OneStep in a clinical population.

Validation experiments were conducted at the Cal Poly Mobile Biomechanics Laboratory (MBL). The 10-camera motion analysis system (Motion Analysis Corp., Santa Rosa, CA, USA) was calibrated to establish a global coordinate system. Marker data from the motion analysis system were sampled at 200 Hz. The difference in sampling frequencies between the OneStep app and MoCap system were deemed acceptable when comparing gait variables for this study, as both systems’ frequencies were high enough to capture changes in gait variables.

The first phase of the walking trials was conducted in the MBL with the MoCap system and smartphone simultaneously. Participants wore compressive clothing during data collection. Three retroreflective markers were attached to the participant: one on the sacrum and two on the calcanei of the feet. Participants were asked to remove their shoes and socks to mitigate heel marker translation during data collection.

**Figure 1:**
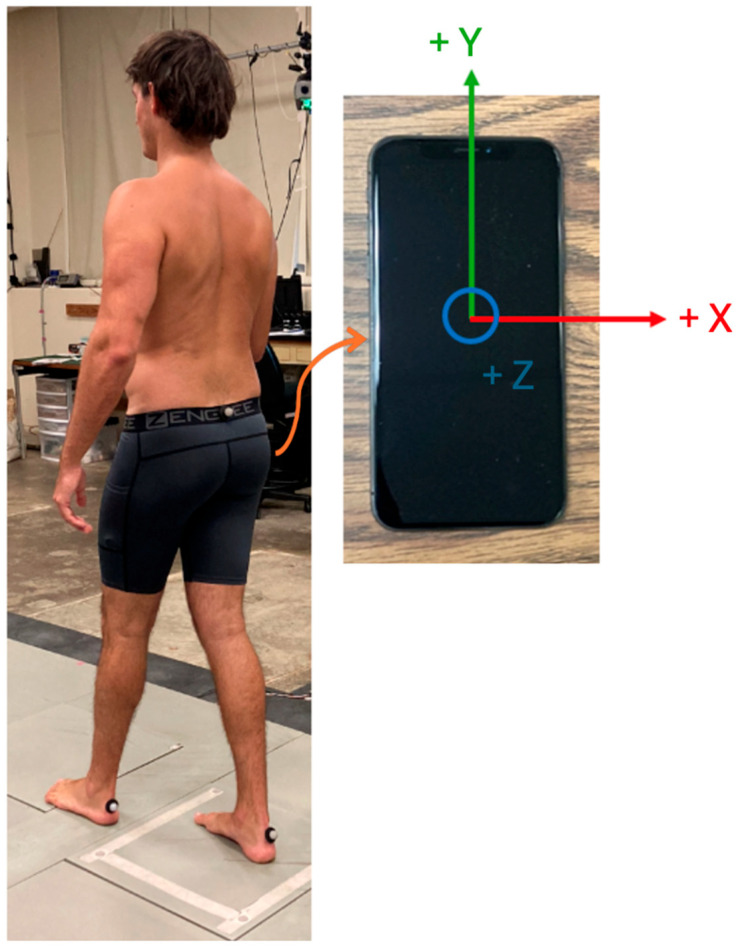
Photo of participant performing walking trial in MBL (**left**) and photo of smartphone axes’ orientation and location during trials (**right**). Phone was placed in right pocket of participant.

Participants were instructed to perform three walking trials at their natural pace while performing the serial seven test (counting backwards from 100, subtracting 7 each time) along the ~35-foot walkway in the MBL (Figure 1). The purpose of the serial seven test was to provide a cognitive challenge during the trial and to prevent the Hawthorne effect (awareness of being evaluated or presence of an observer, leading to potentially influencing one’s gait) [12]. A successful trial in the MBL was defined as when the OneStep app yielded a gait report for each recording, which requires a minimum of 20 steps.

For the second phase of the validation experiments, three 1-min walking trials were performed in the hallway of the lab’s building, with participants wearing their normal footwear and using the OneStep app on the smartphone. Because human gait is known to vary with context (such as walkway characteristics and footwear) [13,14,15], this phase was not intended as a device-validation test but rather to characterize environment-related differences in gait metrics when using OneStep in real-world clinical hallways settings and to verify that, despite expected differences in absolute values, participants’ relative gait patterns were preserved. Participants performed these walking trials at a self-selected pace while completing the serial seven test.

### 2.3. Data Processing for Validation Study

#### 2.3.1. Data Smoothing and Averaging

Cortex data were exported from the MoCap system and were smoothed using a 6 Hz cutoff, zero-phase, 4th-order Butterworth filter. For validation experiments, all OneStep gait parameters were averaged between all three trials of each participant. Gait parameters calculated using Cortex data are displayed as an average of one trial. For in-clinic experiments, all OneStep gait parameters were averaged between all three trials for all three appointments.

#### 2.3.2. MATLAB Algorithms

The motion analysis data were imported into MATLAB R2022a (MathWorks, MA, USA). The following sections explain how each gait variable was calculated using heel and sacrum marker data, and Table 1 summarizes the equations used.

##### Stride Length (StdL)

Heel strike (HS) times were identified as the maximum peaks when taking the difference between sacrum and heel position in the X direction [16]. For each foot, subsequent peaks, HS_i+1_ were subtracted from the precedent peak, HS_i_. The resultant values of heel strikes (HS) from both feet were averaged together to calculate stride length.

##### Step Length (StpL)

One length of a step was calculated by subtracting subsequent heel strike position events of both feet, HS. The resultant values of HS were averaged to calculate average step length.

##### Step Width (SW)

Inter-marker distance between heel markers was defined as the lowest resultant position between markers over time, where X_X_ is position of the marker in the X direction, X_Y_ is the position of the marker in the Y direction, and X_Z_ is the position of the marker of in the Z direction relative to the global coordinate system. Local minima peaks were averaged to calculate average step width.

##### Cadence (C)

The number of HS events that occurred between the initial HS and final HS of a trial were counted, and their corresponding event times to define n_steps_ and t_steps_, respectively, were used to calculate average cadence.

##### Gait Velocity (GV)

The averaged absolute value of sacrum position in the X direction, $\bar{x}$_sacrum_ over the whole trial, t_sacrum_, was used to calculate average gait velocity.

##### Double Stance Time (DS)

Toe-off (TO) events were identified using MATLAB’s findpeaks function and heel marker acceleration data [17]. HS and TO events of both feet, HS_L_ and HS_R_, were ordered chronologically. The difference of the sum of all TO and HS events over the time of one gait cycle, HS_100%_, HS_0%_, were used to calculate average double stance time.

### 2.4. In-Clinic Experiment Methods

Eleven patients (5M/6F, aged 70.91 ± 6.56 years, 8 right leg dominant and 3 left leg dominant) were recruited from Central Coast Orthopedic Clinics in San Luis Obispo and Pismo Beach, California. The inclusion criteria for clinical experiments were adults who were undergoing a primary TKA between September 2024 and March 2025 and who were English-speaking. The exclusion criteria included history of cardiovascular, respiratory, or any other metabolic disease/complications that would inhibit one’s ability to walk down a hallway, and any lower extremity injury within the past 6 months. There was no grouping of participants based on participant demographics or otherwise.

Walking trials were conducted at three timepoints: (1) pre-operative appointment, (2) approximately 2-weeks post-operative appointment, and (3) approximately 6-weeks post-operative appointment. These time points were conveniently chosen to align data collection with the patients’ standard post-operative appointment dates with the clinic. Additionally, previous literature has identified significant and meaningful recovery after TKA starting at 4-weeks post-TKA, which was used as a marker for this study to observe functional decline at 2-weeks post-TKA and improvement at 6-weeks post-TKA [4,9]. Participants were allowed to use assistive devices if needed during their post-operative appointments to ensure safety during walking.

Three 1-min walking trials were conducted in the hallway of the clinic using the OneStep app. Similar to the validation studies, the phone was placed in their pocket or in the phone pouch at the pocket location. During each trial, participants were asked to walk at a natural pace and complete the serial seven test.

### 2.5. Statistics

Statistical analyses were conducted to (1) validate OneStep against a traditional MoCap system, (2) provide insight into OneStep’s ability to detect TKA post-operative recovery trends, and (3) evaluate differences in gait variables pre-operatively and post-operatively to assess patterns of functional recovery after TKA.

Paired *t*-tests, coefficients of determination, and linear regressions between OneStep and MoCap data and between walking conditions were used to address the first objective using the validation experiments. For the linear regressions, the gold-standard MoCap system was designated as the independent variable and OneStep as the dependent variable. Shapiro–Wilks tests were used to assess data normality for gait variables between methods and between walking conditions. All non-normal data distributions were evaluated using Wilcoxon Rank tests.

Repeated-measures ANOVA tests were used to address the second objective (detecting recovery trends) using the clinic experiments. Gait variables were compared between timepoints and were tested for normality using Shapiro–Wilks tests. Gait variables that violated normality were evaluated using Friedman tests. Post hoc Dunn’s pairwise tests were performed on statistically significant groups, and a Bonferroni correction of 3 (adjusted *p*-value of 0.0167) was used to indicate statistically significant differences in gait variables between timepoints. A significance level of α = 0.05 was used for all statistical tests.

## 3. Results

### 3.1. Validation Study Results

**Figure 2:**
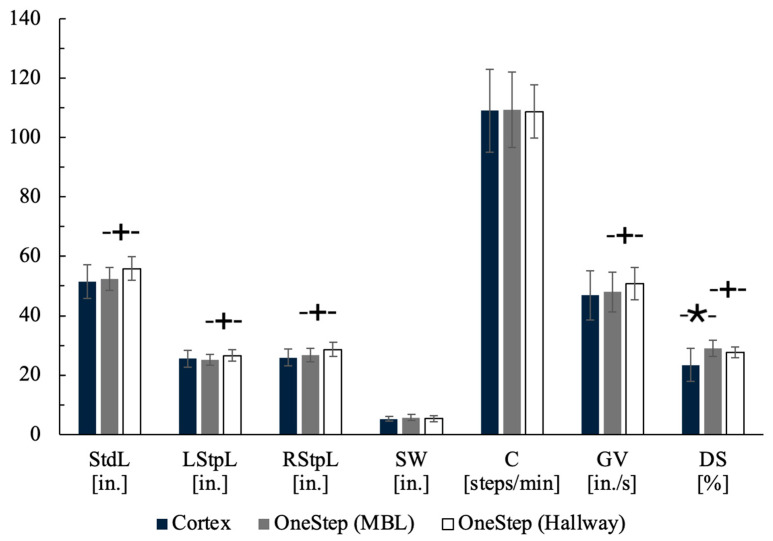
Bar graph of means and standard deviations of Cortex and OneStep values in the MBL and hallway. * indicates statistical significance between methods from *t*-test *p*-value results. + indicates statistical significance between walking conditions from *t*-test *p*-value results. Note: Units for GV are shown in in./s (vs. m/s in other sections) to improve scaling and interpretation of bars.

Means and standard deviations of Cortex and OneStep values in the MBL and hallway are illustrated in Figure 2. Double stance time was the only variable that differed significantly between OneStep (MBL) vs. Cortex (29.02 ± 2.70% vs. 23.40 ± 5.59%, *p* = 0.001). Between conditions (MBL vs. Hallway), significant differences were seen in stride length (52.41 ± 3.80 in. vs. 55.85 ± 4.01 in., *p* < 0.0001), left step length (25.07 ± 1.81 in. vs. 26.61 ± 2.00 in., *p* < 0.0001), right step length (26.76 ± 2.19 in. vs. 28.61 ± 2.38 in., *p* < 0.0001), gait velocity (48.03 ± 6.69 in./s vs. 50.79 ± 5.51 in./s, *p* = 0.048), and double stance time (29.02 ± 2.7% vs. 27.65 ± 1.72%, *p* = 0.021). Table 2 provides further details of data normality results for all gait variables between methods and between walking conditions. Markers indicate relationship strength of variables between methods and between walking conditions.

Shapiro–Wilks results between methods and between walking conditions are illustrated in Table 2. Data normality was observed for all gait variables except for cadence between walking conditions (*p* = 0.041), and for double stance time between methods (*p* = 0.00030) and between walking conditions (*p* = 0.040).

Paired *t*-tests, coefficients of determination, and linear regression tests performed on normally distributed gait variables between methods are summarized in Table 3. Paired *t*-test results show high *p*-values, which indicate no statistical difference in gait variables between methods. Additionally, low linear regression *p*-values were observed for all gait variables except step width (*p* = 0.67), indicating a strong relationship of gait variables between methods.

Paired *t*-tests, coefficients of determination, and linear regression tests performed on normally distributed gait variables between walking conditions are summarized in Table 4. Paired *t*-tests indicated expected differences between the controlled lab environment and the hallway condition for all gait variables except for step width (*p* = 0.25) and cadence (*p* = 0.57), consistent with known context-dependent variations in spatiotemporal gait [13,14]. Despite these differences, the metrics remained strongly linearly related across conditions, as indicated by significant *p*-values for linear regression, indicating that participants’ relative gait patterns were preserved. Thus, differences reflect environmental effects on gait rather than measurement inconsistency of the OneStep system.

### 3.2. In-Clinic Experiment Results

Recordings of OneStep gait metrics at each appointment were tabulated. Of the 11 participants, 1 participant was removed from the dataset due to corrupted data files during a post-operative visit. Data normality results from Shapiro–Wilks tests between all timepoint pairs are illustrated in Table 5. All variables except for step width violated normality for at least one timepoint pair.

Table 6 includes means and standard deviations of participants at each appointment, along with *p*-values for ANOVA and Friedman tests. Repeated-measures ANOVA was used for step width due to its normal distribution, while Friedman tests were used for all non-normally distributed variables. All gait variables besides step width showed statistically significant differences between at least one timepoint pair.

Post hoc Dunn pairwise comparisons with a Bonferroni correction (*p* = 0.0167) to account for false positives were performed in response to the Friedman test results (*p* < 0.05). The post hoc Dunn tests revealed that between pre-operative and 2-weeks post-operative (−2 to 2), gait velocity showed statistical significance (*p* = 0.011). Between 2-weeks postoperative and 6-weeks post-operative, stride length (*p* = 0.005) and gait velocity (*p* = 0.005) showed statistical significance. The other variables and timepoints were statistically similar (*p* > 0.022 for all).

**Figure 3:**
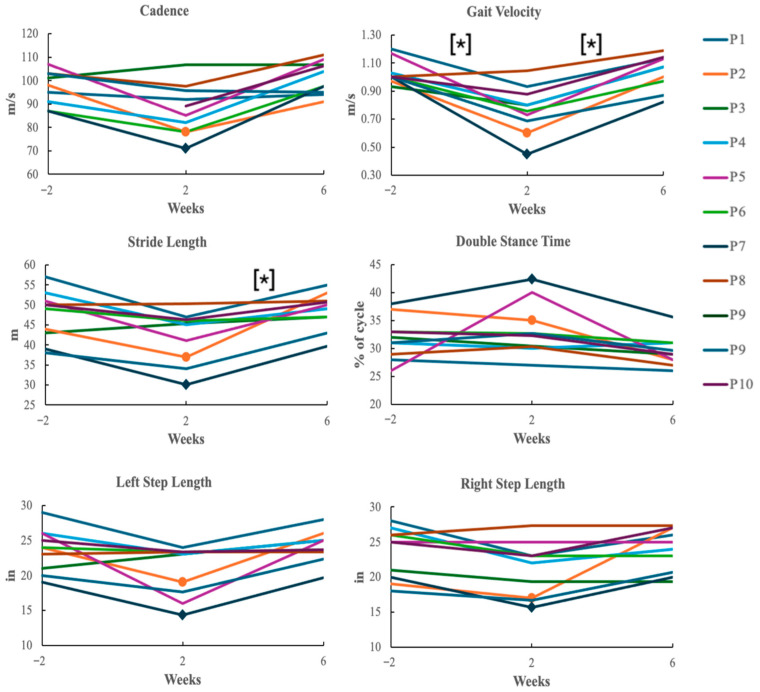
Line graphs of functional recovery of patients at each appointment. Step width is not shown due to a lack of trends observed between timepoints. * indicates statistical significance between weeks based on Post hoc Dunn results. • indicates participants who used a cane during walking trial. ♦ indicates participants who used a walker during walking trial.

Line graphs of gait variables measured at each timepoint are illustrated in Figure 3 to observe functional recovery trends of patients pre- and post-operatively, with significant differences from post hoc Dunn comparisons indicated with asterisks. Data that were identified as outliers were removed from the graphs. Between the pre-operative appointment and 2-weeks post-operative appointment, there were noticeable decreases in stride length, left step length, right step length, cadence, and gait velocity, in addition to an increase in step width and double stance time. Between 2-weeks post-operative and 6-weeks post-operative, the majority of participants had exceeded their 2-weeks post-operative measurements and had met or exceeded their pre-operative gait measurements.

## 4. Discussion

### 4.1. Key Findings

Overall, the findings of this study support the hypotheses that OneStep has shown validity and reliability compared to a MoCap system, and that meaningful differences in stride length and gait velocity for TKA patients pre- and post-operatively were observed using the OneStep app.

OneStep proved to be a valid and reliable alternative to a MoCap system for measuring most of the assessed gait metrics (stride length, left and right step length, cadence, gait velocity), as shown by the statistical similarities in gait metrics between methods in the validation study. Cadence showed the highest similarity between methods (*t*-test: *p* = 0.84; linear regression: *p* < 0.0001). Double stance time was the only variable that violated normality and indicated differences between methods (*t*-test: *p* = 0.0013) and between walking conditions (*t*-test: *p* = 0.021). Although step width was not significantly different between methods based on the *t*-test (*p* = 0.22), regression showed a lack of significant correlation between methods (*p* = 0.67). It is sensible for step width and double stance time to have the poorest performance, since both variables require data from both feet, while OneStep measures data from the one limb where the phone is placed in the pocket.

Clinically, measuring gait variables using OneStep allowed us to assess the functional recovery trends of patients following TKA. Participants’ gait health generally decreased from pre-operative to 2-weeks post-operative and then met or surpassed their pre-operative values at 6-weeks post-operative. Friedman test results showed that there were statistical differences for all gait variables. Post hoc Dunn tests with a Bonferroni correction (*p* = 0.0167) showed statistical differences for stride length when comparing between 2-weeks post-operative to 6-weeks post-operative (*p* = 0.005) and for gait velocity when comparing pre-operative to 2-weeks post-operative (*p* = 0.011) and 2-weeks post-operative to 6-weeks post-operative (*p* = 0.005), but did not reveal statistically significant pairwise differences for step length, cadence, and double stance time. These trends of a decrease around 2-weeks post-operative and improvement around 6-weeks post-operative align with several studies showing similar post-TKA gait recovery trends [9,11,18,19,20], including one study that passively collected gait measurements of participants from pre-operative to 24-months post-operative using a smartwatch paired to a smartphone [9]. Additionally, the OneStep validation results of this study align with another study that assessed the validity of the OneStep app against a validated pressure-sensor walkway among patients with musculoskeletal pathologies [5].

### 4.2. Study Limitations

Given that step width and double stance time require data from both feet, along with methodological limitations of calculating step width and double stance time using only heel marker data, these may have contributed to reduced accuracy in calculating step width and double stance time from both the OneStep application and the motion capture system. However, the OneStep application overall proved to be an inexpensive and more accessible alternative to traditional motion capture for TKA patients and can be utilized as a secondary continuous measurement of recovery in addition to traditional clinical assessments. Mobile methods of calculating clinical gait assessments would likely need to include sensors on both legs.

Another limitation is that while the gait algorithms created in MATLAB from the motion analysis data were validated against similar algorithms in the MBL in previous studies, they were not previously validated against a “ground truth” such as video analyses. For one, the method used to calculate HS and TO events used marker data instead of force data. HS events were identified using an equation that used heel and sacrum marker data [16], while TO events were identified using heel marker acceleration data in the anteroposterior direction [17]. Additionally, double stance time did not account for the numerical differentiation that the Cortex motion analysis software performs on the acceleration data. While it is unknown exactly what type of differentiation Cortex utilizes, differentiation results in small errors in the accelerations that can impact the calculated double stance times. Future work validating double stance time in the motion analysis lab should use force plates to specifically measure times of HS and TO events.

Another limitation of the validation experiments was that young, healthy adults were used to validate the OneStep app, reflecting the typical participant pool available at Cal Poly. While this population limits variability compared to older adults undergoing TKA, the goal of the validation phase was to establish the measurement accuracy of OneStep relative to MoCap under controlled conditions, rather than to generalize directly to the clinical population. Initial validation in healthy walkers is standard practice for wearable gait technologies ([4,21]), as it enables precise and consistent comparison against a gold standard. Once accuracy was confirmed, OneStep was used in a consistent hallway environment to evaluate within-subject pre/post-operative changes in TKA patients. Although future work should include direct validation in populations with abnormal gait, the present approach represents an appropriate first step. A limitation of the OneStep app itself is that the gait variables calculated by OneStep’s algorithm have low precision (only to 1 decimal point), resulting in OneStep values reporting values with a lower resolution than that of the MoCap system.

A clinical limitation was that two of the eleven participants used assistive devices such as canes or walkers post-operatively, resulting in possibly capturing an altered version of their gait compared to their pre-operative gait without an assistive device, thus affecting interpretation of recovery trajectories. However, the use of assistive devices (if needed) was required to ensure the safety of patients during walking trials.

### 4.3. Future Directions

For OneStep validation in MBL, future work should first prioritize evaluating this study’s methods against a “ground truth” for all gait variables. Additional supplementary methods could be used to evaluate step width and double stance time values in patients, since OneStep’s values of these variables were found to have low accuracy. For example, step width could be calculated using synchronous video capture with image processing during walking trials, and double stance time could be calculated by collecting force plate data during walking trials. Furthermore, other markerless motion capture methods such as OpenCap (Stanford, CA, USA), which uses the smartphone’s camera feature to calculate spatial and temporal variables, could be validated for TKA patients and may have higher accuracy, but is a less accessible option than OneStep. Future work should then focus on validating OneStep in populations with abnormal gait patterns for application towards broader clinical applications.

Future work using the OneStep app should continue to follow patients beyond the 6-weeks timepoint set for this study and should assess effectiveness of post-operative recovery methods such as physical therapy. Patients should be followed until their post-operative gait values meet or exceed their pre-operative gait values (typically ranging from 6 months to 2 years). Following patients would further bolster a smartphone’s reliability to measure gait values over time when using apps such as OneStep. Additionally, future studies will involve larger cohorts of patients to increase power. While this study’s sample size reflected recruitment constraints, subsequent studies should perform an a priori power analysis to determine the sample size needed to achieve 80–90% power for detecting clinically relevant gait differences.

Future directions for using the OneStep app in clinical experiments could involve evaluating the pace of recovery between surgical interventions such as a mechanical vs. kinematic knee alignment, or different approaches to the surgical intervention. OneStep may be able to provide insight on which intervention yields the fastest pace of recovery for patients.

### 4.4. Overall Conclusion

This study contributes to the growing literature of using smartphone-based gait analysis, especially in an under-researched population such as those undergoing TKA. The demonstrated validity and reliability of the OneStep app determined from the validation group of this study emphasizes the ease and accessibility of monitoring one’s gait health without the use of a traditional motion capture system. Furthermore, implementing the OneStep app as a secondary measurement tool alongside traditional clinical gait assessments uniquely benefits the TKA population due to the convenience of the app and the ability of patients to monitor their recovery post-TKA.

## Figures and Tables

**Table 1:** Equations used to calculate each gait variable.

Variable	Equation
Stride length	$StdL=HS_{i+1}-HS_{i}$
Step length	$StpL=HS_{left/right}-HS_{right/left}$
Step width	$SW=\sqrt{{\left({x_{X}}_{left}-{x_{X}}_{right}\right)}^{2}+{\left({x_{Y}}_{left}-{x_{Y}}_{right}\right)}^{2}+{\left({x_{Z}}_{left}-{x_{Z}}_{right}\right)}^{2}}$
Cadence	$C=\frac{n_{steps}}{t_{steps}}$
Gait velocity	$GV=\left|\frac{{\bar{x}}_{sacrum}}{t_{sacrum}}\right|$
Double stance time	$DS=\frac{\left(TO_{R}-HS_{L}\right)+\left(TO_{L}-HS_{R}\right)}{HS_{100\%}-HS_{0\%}}=\frac{\left(TO_{L}+TO_{R}\right)-\left(HS_{L}+HS_{R}\right)}{HS_{100\%}-HS_{0\%}}$

**Table 2:** Shapiro–Wilks test *p*-values for gait variables between methods and between walking conditions. * indicates statistical significance.

	Gait Variables						
	StdL	LStpL	RStpL	SW	C	GV	DS
Methods	0.071	0.25	0.10	0.84	0.57	0.63	0.00030 *
Walking conditions	0.90	0.49	0.34	0.49	0.041 *	0.26	0.040 *

**Table 3:** Statistical test results for reliability of gait variables between methods (Cortex vs. OneStep). Group comparison *p*-values are from paired *t*-tests for normally distributed variables and Wilcoxon Rank tests for non-normally distributed variables. * indicates statistical significance.

	Gait Variables						
	**StdL**	**LStpL**	**RStpL**	**SW**	**C**	**GV**	**DS**
*p*-value (group comparisons)	0.42	0.48	0.17	0.22	0.84	0.31	0.0013 *
$R^{2}$	0.32	0.24	0.27	0.013	0.90	0.67	--
*p*-value (regression)	0.019 *	0.047 *	0.034 *	0.67	<0.0001 *	<0.0001 *	--

**Table 4:** Statistical test results for reliability of gait variables between walking conditions (MBL vs. Hallway). Group comparison *p*-values are from paired *t*-tests for normally distributed variables and Wilcoxon Rank tests for non-normally distributed variables. * indicates statistical significance.

	Gait Variables						
	StdL	LStpL	RStpL	SW	C	GV	DS
*p*-value (group comparisons)	<0.0001 *	<0.0001 *	<0.0001 *	0.25	0.57	0.048 *	0.021 *
$R^{2}$	0.67	0.69	0.77	0.35	0.70	0.52	--
*p*-value (regression)	<0.0001 *	<0.0001 *	<0.0001 *	0.0127 *	<0.0001 *	<0.0001 *	--

**Table 5:** Shapiro–Wilks test results for gait variables between pre-operative and 2-weeks post-operative appointments (−2 to 2), 2-weeks post-operative and 6-weeks post-operative appointments (2 to 6), and pre-operative and 6-weeks post-operative appointments (−2 to 6). * indicates nonnormally distributed gait variables.

	Gait Variables						
Comparisons	StdL	LStpL	RStpL	SW	C	GV	DS
−2 to 2	−10.97 *	−12.66 *	−9.79 *	−3.17	−7.01	−25.50 *	4.63
2 to 6	15.01 *	16.75 *	12.89 *	1.09	15.70 *	35.45 *	11.54 *
−2 to 6	2.39	1.97	1.84	−2.12	7.58	0.91	−7.44 *

**Table 6:** Means and standard deviations of gait variables at each appointment. *p*-values were obtained using repeated-measures ANOVA for step width (normal distribution) and Friedman tests for all other variables (non-normal distributions). * indicates statistical significance.

	Gait Variables						
Weeks	StdL (in.)	LStpL (in.)	RStpL (in.)	SW (in.)	C (Steps/min)	GV (m/s)	DS (%)
−2	47.40	23.70	23.50	6.30	94.10	1.03	31.80
	±6.17	±3.06	±3.63	±1.16	±11.20	±0.09	±3.74
2	42.20	20.70	21.20	6.10	87.50	0.77	33.27
	±6.52	±3.61	±3.87	±1.14	±10.80	±0.17	±4.70
6	48.53	24.17	23.93	6.17	101.23	1.04	29.43
	±4.58	±2.23	±3.05	±1.24	±6.99	±0.12	±2.71
*p*-value	*p* = 0.0060 *	*p* = 0.012 *	*p* = 0.019 *	*p* = 0.90	*p* = 0.023 *	*p* = 0.0020 *	*p* = 0.012 *

## Data Availability

Please direct data inquiries to Britta Berg-Johansen at bbergjoh@calpoly.edu. Some raw data may be unavailable due to participant confidentiality agreements.

## References

[1] Hsu H., Siwiec R.M. (2023). Knee Osteoarthritis. StatPearls.

[2] Marino G., De Capitani F., Adamo P., Bolzoni L., Gatti R., Temporiti F. (2024). Long-term gait analysis in patients after total knee arthroplasty: A systematic review and meta-analysis. Gait Posture.

[3] Hsu H., Siwiec R.M. (2023). Knee Arthroplasty. StatPearls.

[4] Christensen J., Stanley E., Oro E., Carlson H., Naveh Y., Shalita R., Teitz L. (2022). The validity and reliability of the OneStep smartphone application under various gait conditions in healthy adults with feasibility in clinical practice. J. Orthop. Sports Phys. Ther..

[5] Shema-Shiratzky S., Beer Y., Mor A., Elbaz A. (2022). Smartphone-based inertial sensors technology—Validation of a new application to measure spatiotemporal gait metrics. Gait Posture.

[6] Matikainen-Tervola E., Cronin N., Aartolahti E., Sihvonen S., Sansgiri S., Finni T., Mattila O., Rantakokko M. (2024). Validity of IMU sensors for assessing features of walking in laboratory and outdoor environments among older adults. Gait Posture.

[7] Werner C., Hezel N., Dongus F., Spielmann J., Mayer J., Becker C., Bauer J. (2023). Validity and reliability of the Apple Health app on iPhone for measuring gait parameters in children, adults, and seniors. Sci. Rep..

[8] Brognara L. (2024). Gait Assessment Using Smartphone Applications in Older Adults: A Scoping Review. Geriatrics.

[9] Fary C., Cholewa J., Abshagen S., Andel D.V., Ren A., Anderson M.B., Tripuraneni K.R. (2023). Stepping beyond Counts in Recovery of Total Knee Arthroplasty: A Prospective Study on Passively Collected Gait Metrics. Sensors.

[10] Getting Raw Accelerometer Events. https://developer.apple.com/documentation/coremotion/getting-raw-accelerometer-events.

[11] Gianzina E., Kalinterakis G., Delis S., Vlastos I., Sachinis N., Yiannakopoulos C. (2023). Evaluation of gait recovery after total knee arthroplasty using wearable inertial sensors: A systematic review. Knee.

[12] Jeon J., Kwon S., Lee Y., Hong J., Yu J., Kim J., Kim S., Lee D. (2023). Influence of the Hawthorne effect on spatiotemporal parameters, kinematics, ground reaction force, and the symmetry of the dominant and nondominant lower limbs during gait. J. Biomech..

[13] Lordall J., Oates A., Lanovaz J. (2024). Spatiotemporal walking performance in different settings: Effects of walking speed and sex. Front. Sports Act. Living.

[14] Zoppo C., Belluscio V., Vannozzi G. (2025). Impact of Walking Path Length on Gait Parameters During the 2-Minute Walk Test in Healthy Young Adults. Biomechanics.

[15] Eggleston J., Conroy K., Moreno A., Travis W., Huskey B., Vanderhoof H. (2023). The use of preferred footwear versus barefoot conditions in gait analysis: A methodological investigation. J. Biomech..

[16] Zeni J., Richards J., Higginson J. (2008). Two simple methods for determining gait events during treadmill and overground walking using kinematic data. Gait Posture.

[17] Determining Initial-Contact & Toe-Off from Kinematic Data Alone. http://www.clinicalgaitanalysis.com/faq/toe-off.html.

[18] Cushner F., Yergler J., Elashoff B., Aubin P., Verta P., Scudero G. (2025). Staying Ahead of the Curve: The Case for Recovery Curves in Total Knee Arthroplasty. J Arthroplast..

[19] Szczypiór-Piasecka K., Adamczewska P., Kołodziej Ł., Ziętek P. (2025). The Temporal–Spatial Parameters of Gait After Total Knee Arthroplasty. J. Clin. Med..

[20] Gu X., Zhou C., Zhu X., Cao J., Li H. (2025). Early postoperative gait characteristics after unicompartmental knee arthroplasty: Results and clinical implications. BMC Musculoskelet. Disord..

[21] Tao S., Zhang H., Kong L., Sun Y., Zhao J. (2024). Validation of gait analysis using smartphones: Reliability and validity. Digit. Health.

[22] Thai C.A. (2024). Measuring Gait of Total Knee Arthroplasty Patients Pre-Op and Post-Op Using a Smartphone. Master’s Thesis.

